# Unusual Lesion in the Splenium of the Corpus Callosum and COVID-19 Infection: A Case Report

**DOI:** 10.5334/jbsr.2901

**Published:** 2023-02-06

**Authors:** Wiem Abid, Tim Vanderhasselt, Gert-Jan Allemeersch

**Affiliations:** 1 UZ Brussel, BE

**Keywords:** SARS-CoV-2, The splenium of the corpus callosum, CLOCCS, Magnetic Resonance Imaging, Cytokinopathy, Glutamate Excitotoxicity

## Abstract

The novel coronavirus (SARS-CoV-2) causing the recent pandemic outbreak may result in brain injuries. The disease has a high prevalence for thromboembolic complications and a massive release of cytokines. We report a case of CLOCCS, one of the rare neurological complications of SARS-CoV-2 infection.

**Teaching Point:**

The imaging features of the cytotoxic lesion of the corpus callosum (CLOCCS) on magnetic resonance imaging should be known by every radiologist, to make the positive diagnosis and prevent misdiagnosis, especially in the setting of a COVID-19 infection.

## Introduction

The cells of the corpus callosum (CC) have a high density of cytokine and glutamate receptors. The activation of microglia in case of infection leads to a cytokinopathy with release of Glutamate. A Glutamate excitotoxicity process is initiated leading to cytotoxic edema that manifests as reduced diffusion at magnetic resonance imaging (MRI) [1].

## Case History

A 19-year-old woman, with no medical history, presented in the emergency department with altered mental status, lethargy, and acute confusion. She had symptoms of holocranial headache, vomiting and photophobia. She had no fever. The patient had an altered Glasgow Coma Score (GCS) of 13/15 with a normal neurological examination. A polymerase chain reaction (PCR) from a nasopharyngeal swab was positive for SARS-CoV-2 virus infection. The patient was not vaccinated for SARS-CoV-2 virus and had no signs of severe acute respiratory syndrome coronavirus. A cerebral computed tomography (CT) scan was normal. A cerebrospinal fluid in the case history (CSF) analysis and an electroencephalogram (EEG) examination were normal. Three days later, the evolution was complicated by the development of a severe akinetic mutism.

**Figure 1:**
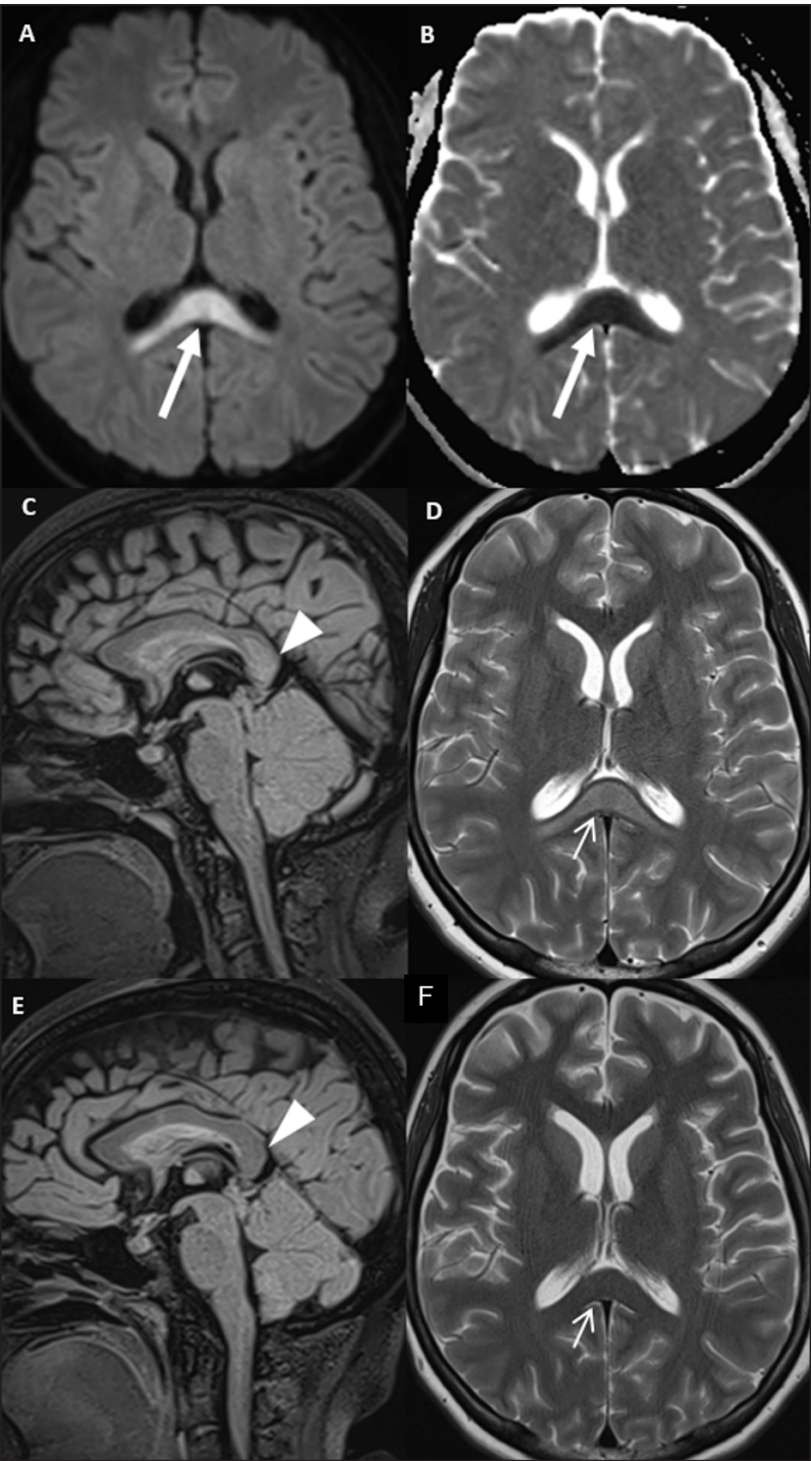
Axial DWI and ADC images **A** and **B** showed diffusion restriction in the splenium of the CC (long arrows showing high signal intensity on DWI on image **A** and low signal intensity on ADC on image **B**) with a high signal intensity on the sagittal FLAIR on image **C** (arrowhead showing high signal intensity in the splenium) and a high signal intensity on the axial T2 on image **D** (short arrow showing high signal intensity in the splenium) suggesting a CLOCCS. Sagittal FLAIR on image **E** (arrowhead showing the disappearance of the high signal intensity in the splenium) and axial T2 on image **F** (short arrow showing the disappearance of the high signal intensity in the splenium) showed a complete resolution of the CLOCCS signs one week later.

**Figure 2:**
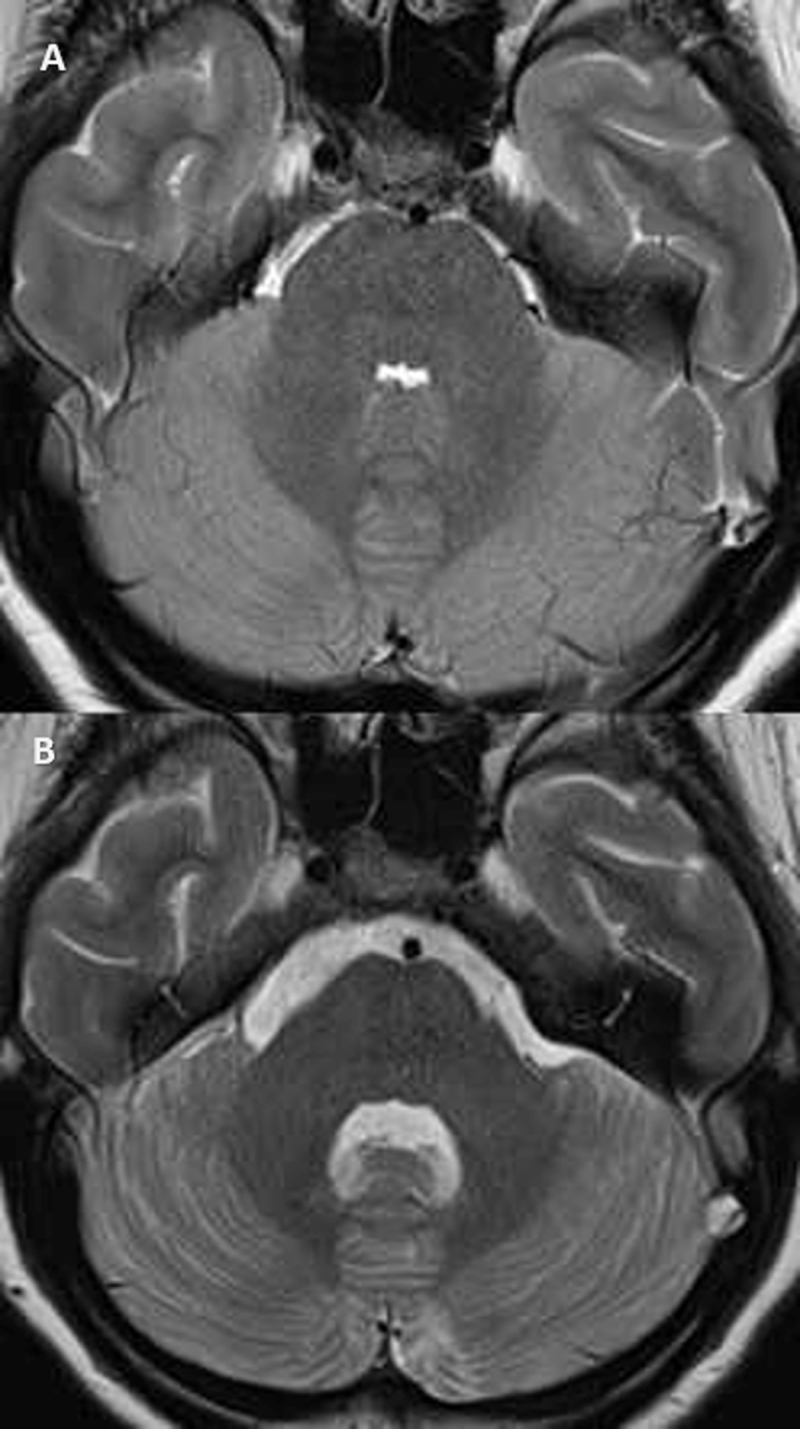
Axial T2 on image **A** showed an associated cerebellitis with a slight high signal intensity of the parenchyma and a mass effect on the fourth ventricle and on the basal cisterns. Axial T2 on image **B** depicted a decrease of the cerebellitis signs one week later with a normalization of the signal intensity of the parenchyma and a decrease of the mass effect on the fourth ventricle and on the basal cisterns.

Magnetic resonance imaging (MRI) of the brain performed on the same day depicted a cerebellitis with edema and diffuse and symmetric T2/FAIR high signal intensity zones of the cerebellum with gadolinium enhancement and massa effect on the fourth ventricle (Figure 2A). No bleeding was depicted. Diffusion restriction in the splenium of the corpus callosum was seen with a slightly high signal intensity on FLAIR (Figure 1A–D). The diagnosis of cerebellitis and cytotoxic lesions of the corpus callosum (CLOCCS) was made. The evoked differential diagnosis was an ischemic lesion of the corpus callosum post COVID-19 infection.

One week later, the MRI control showed a complete resolution of the CLOCCS signs (Figure 1E–F) with a regression of the cerbellitis signs (Figure 2B).

## Comments

Splenium lesions with reversible or irreversible damage may be encountered in many neurologic and non-neurologic diseases [2, 3]. The CC is the largest thick commissural white matter bundle in the brain [3]. The splenium is the posterior part of the CC [2]. Unlike the major portion of the CC receiving its arterial supply from the carotid system, the splenium is supplied by the vertebrobasilar system [3].

Lesions of the splenium with signal changes seem to be nonspecific and could be seen in different conditions from various etiologies and pathogenic mechanisms. They should not be confused with serious pathologies [3]. It is important to differentiate between the primary and the secondary callosal lesions.

**Primary lesions** include arterial occlusion resulting in ischemic infarction of the CC, acute disseminated encephalomyelitis (ADEM), multiple sclerosis with typical periventricular white matter lesions and tumoral lesions such as lymphoma or glioblastoma with a more aggressive appearance. In this case enhancement on MRI may help in the diagnosis when the imaging findings are equivocal [1].

**Secondary lesions** are represented in CLOCCS. CLOCCS are nonspecific and have been reported in association with many conditions, for instance in patient with seizures, in drug therapy, malignancy, infections, subarachnoid hemorrhage, metabolic disorders, trauma [1].

Signal changes of the splenium on MRI showing diffusion restriction in case of CLOCCS depend on the shape and the extent: the first type can manifest with an oval circumscribed zone, with well-defined borders usually located in the middle; the second type can be wider, with less regular borders and involving the entire splenium (‘Boomerang sign’) [3].

Microglia, which are the macrophages of the central nervous system (CNS), and monocytes become active in case of infection, trauma or inflammation and release the inflammatory cytokines interleukin 1 (IL-1) and IL-6 leading to a cytokinopathy and a breakdown of the blood-brain barrier. Astrocytes stimulated by IL-1 release glutamate. Its extracellular concentration is increased. Glutamate has an excitotoxic action on receptors, sodium-potassium pumps, and aquaporins. The influx of water into both astrocytes and neurons increases secondary with installation of intracellular edema and reduction of the diffusion known as cytotoxic edema. Neurons, astrocytes, and oligodendrocytes of the CC have a higher density of cytokine and glutamate receptors [1].

To make the specific diagnosis and find the underlying cause, associated imaging findings should be depicted with a clinical correlation. A misdiagnosis of ischemia should not be made [1]. The absence of symptoms of hemispheric disconnection and reversibility after control of underlying disease is suggestive of CLOCCs. Moreover, primary lesions can show contrast enhancement, which is very rarely present with CLOCCS [3]. Those lesions are generally reversible, and reversibility indicates a better prognosis [2].
